# Pulmonary Tuberculosis

**Published:** 1905-09-23

**Authors:** 


### PULMONARY TUBERCULOSIS.

Dr. H. P. Loomis,[1] contributes to the July issue of the *Medical Record* the results of an interesting study of 500 cases of pulmonary tuberculosis. The study was conducted along the following four lines: (1) Inquiry as to the very earliest symptoms of pulmonary tuberculosis, from an analysis of 100 cases; (2) an analysis of 55 cured cases of the disease; (3) a retrospective consideration of the bases of prognosis; (4) a study of the average duration of life in the case of the poor in great cities who are attacked by the disease.

1. Dr. Loomis considers that in reviewing the histories of onset of pulmonary tuberculosis it is necessary to catalogue the evidence under two heads—(*a*) the first presumptive evidence; and (*b*) the first demonstrable evidence of the infection. He selected 100 cases where a full and careful record of the earliest symptoms was supplied, and discovered upon analysing them that in 80 per cent. of them the first presumptive evidences of the malady fell into three groups. (1) Long-continued colds or coughs; (2) Ill-defined loss of weight and strength in the early stages, without cough; (3) pleurisy, whether serous or dry. The average duration of the period of presumptive infection—that is, the period between the onset of ill-health and the frank declaration of the disease was five and a-half months.

Of the 100 cases 14 gave a history of previous pleurisy, while a second series of 260 cases yielded a preliminary pleuritic phase in 44 per cent. of the total patients considered. The average interval between the first attack of pleurisy and the actual declaration of the disease was three and a half years. As to the probability of pulmonary disease after a pleurisy with effusion, it appeared from a consideration of 50 cases that, after an interval of five years, 12 had developed tuberculosis.

The 20 per cent. of the cases remaining after the exclusion of those falling under the three heads above-mentioned, gave histories, with approximately equal frequency, of influenza, malaria, pneumonia, enlargement of the cervical glands, and hæmoptysis. In four cases only did hæmoptysis appear in persons apparently in perfect health. Dr. Loomis holds that hæmoptysis represents a demonstrable lesion in a vast majority of cases, and that where no such evidence is at the time obtainable, it will certainly appear if the cases be watched. He asserts that it is the one incontestable symptom of pulmonary tuberculosis in patients under 30 years of age who have no cardiac or hepatic lesion and who are not "bleeders."

The symptoms which led to the physical demonstration of the lesion were distributed as follows:—(1) In 58, cough. This was associated with expectoration in 42 instances. (2) Hæmoptysis in 24 instances. (3) Fever with night-sweats or chills in 10 instances. The few remaining were ushered in by influenza or tuberculous pneumonia.

The analysis of these cases leads the author to the following conclusions:—(*a*) That the great majority of patients who appear to develop tuberculosis of the lung after their thirtieth year have had a previous attack, though it may not have been recognised in its true light. (*b*) That the average interval between the inception of the disease and the appearance of tubercle bacilli in the sputa is three and a third months. Thus the presence of tubercle bacilli in the sputa is not so early a manifestation as is sometimes believed to be the case.

2. The 55 cases of apparently cured tuberculosis of the lungs were not selected; they were simply those met with in the ordinary course of work, but cases in which full records existed. They were cases discharged during the past two and a half years, and 44 of them had been followed up to the date of writing. Of these 44 all save two were well and about their ordinary business. They were not all early cases, for 26 showed involvement of both lungs. The analysis has revealed the following facts, (1) that the average age of the cured cases was relatively high—namely, 29 years; (2) That in the cured cases a history of longevity in the parents was a remarkably constant feature. Of 47 cured patients who could account for their parents, 31 had both parents surviving after the age of 60. On the other hand, of a similar number living under similar conditions, who did not improve, only 16 had both parents living and over 60. The author is strongly of opinion that the evidence of inherent resistance supplied by the longevity of parents is of the highest value in swaying judgment in the matter of prognosis. He has applied the principle in the case of pneumonia, and with considerable success; (3) That the period of time occupied by the cure, dating from the beginning of climatic treatment, was approximately equal to the period elapsing between the inception of the disease and the beginning of such treatment.

3. A retrospect of the prognoses in 100 cases admitted to a sanatorium, gives the following information. Of the cases considered at the time of examination to be very favourable, 30 per cent. recovered, 50 per cent. improved, and 20 per cent. remained *in statu quo* or became worse. Of the good cases—*i.e.* with a fair outlook, 22 per cent. were cured, 44 per cent. improved, and 33 per cent. remained unimproved or grew worse. Lastly, of the unfavourable cases, 27 per cent. were cured, 36 per cent. improved, and 36 per cent. remained unimproved or grew worse. The comparison of these figures is facilitated by the following tabular expression:—

| | Apparently cured Per cent. | Improved Per cent. | Worse Per cent. |
|---|---|---|---|
| Favourable cases ... | 30 | 50 | 20 |
| Good cases, fair outlook | 22 | 44 | 33 |
| Unfavourable cases ... | 27 | 36 | 36 |

These results impress one with the faultiness of the recognised data from which we are taught to estimate the prognosis in tubercular conditions of the lung. In the light of the above records Dr. Loomis comes to the following conclusions as to prognosis.

(*a*) Evidence of high vitality is a good sign, such as a history of previous freedom from illness, and of longevity in the parents.

(*b*) If it be certain that the disease had its inception between the ages of 25 and 30, the outlook is better than if the patient is attacked at an earlier age.

(*c*) A buoyant sunny temperament is an immense asset in the direction of recovery.

(*d*) Whether one lung, or more than one, is involved, does not appear to affect the prognosis so much as is generally taught.

4. As regards the average duration of life among the poor who fall victims to the disease, Dr. Loomis finds that when the disease is of the usual chronic type the mean duration is one year and two months, and when of the acute type, two months and a half.

[1] Med. Record, N.Y., July 29, 1905.

